# Young’s Modulus Calculus Using Split Hopkinson Bar Tests on Long and Thin Material Samples

**DOI:** 10.3390/ma15093058

**Published:** 2022-04-22

**Authors:** Adrian-Nicolae Rotariu, Eugen Trană, Liviu Matache

**Affiliations:** Military Technical Academy “Ferdinand I”, 39-49 G. Cosbuc Ave., 050141 Bucharest, Romania; adrian.rotariu@mta.ro (A.-N.R.); liviu.matache@mta.ro (L.M.)

**Keywords:** elastic modulus, mechanical impedance mismatch, SHPB test

## Abstract

Young’s modulus is a key parameter of materials. The method of its calculation in the current paper is concerned with the mismatch of the mechanical impedance at the bar/specimen interface for a compression SHPB (split Hopkinson pressure bar) test. By using long and thin specimens, the signal recorded in the transmission bar presents itself as a multistep signal. The ratio between the heights of two successive steps represents the experimental data that are considered in the formula of the elastic modulus this article is devoted to. The oscillatory nature of the real signals on the horizontal or quasi-horizontal segments prevents a precise determination of the two successive step heights ratio. A fine tuning of this value is made based on the characteristic time necessary for the signal to rise from one level to the next one. The FEM (Finite Element Method) simulations are also used in calculation of the Poisson coefficient of the tested complex concentrated alloy.

## 1. Introduction

The modulus of elasticity is an intrinsic property of materials and a key parameter in the design and development of materials. Thus, for engineering applications it is vital to understand this characteristic with high precision in order to both perform finite element calculations or to define constitutive models. However, the test methods currently used, either for static or dynamic regime, have some accuracy issues [1].

The tensile test is the main static method used for the calculus of the elastic modulus and involves the calculation of the stress–strain diagram slope on the region where only elastic deformations are present. The main problems associated with measuring accuracy for this method are related to alignment, deformation measurement, and data analysis. For homogeneous and isotropic materials, Young’s modulus may also be obtained from a static bending test [2]. Another static testing method applicable to thin films is indentation [3,4]. The compression test is not considered a reliable method for the Young’s modulus as long as machine compliance affects the accuracy of the measurements.

For the dynamic regime, a wide variety of testing methods have been developed, including resonance, impact excitation, and acoustic wave propagation methods [5,6,7,8,9,10]. The scattering of the elastic modulus values measured by these methods is usually lower than in the case of static methods and the accuracy is influenced by the possible damages to the samples caused by mechanical processing, by the surface finish quality, and by the dimensional tolerances [1]. Despite the mentioned challenges, the dynamic methods have the advantage to use small-size samples and being relatively fast and simple [1], which makes them suitable for the study of material properties in the development phase. A piece of widely-used equipment for material dynamic behavior research is the split Hopkinson pressure bar (SHPB). However, the SHPB classic configuration does not allow the study of material behavior when small strains, of elastic nature, are induced in samples, which is a prerequisite to study and determine a material’s Young’s modulus. The reason behind this shortcoming is the occurrence of high frequencies harmonics [11] which travel along the SHPB bars with a velocity smaller than the speed of sound [12]. Considerable effort has been made over time to eliminate this shortcoming through various changes in SHPB configuration [13,14,15,16,17,18,19]. The improvements thus obtained paved the way for the use of the SHPB to determine the longitudinal elastic modulus [20,21,22,23]. However, the conclusions regarding the possibility of using simple short cylindrical samples of metal alloys for this purpose are negative [20].

On the other hand, research on thin and excessively long samples of PMMA has shown that a step signal incident pulse becomes a multi-step one in the transmitter bar [23]. This particular form of the signal was used to establish the characteristic time necessary to travel back and forth on the sample, a value later used for sound velocity calculus, respectively, the longitudinal elastic modulus [23]. As shown in [23] the repeated signal jumps recorded in the transmission bar are characterized by a microseconds time rise. Nevertheless, this aspect is not discussed by the authors of the previously-mentioned study in terms of determining the characteristic time used to calculate the Young’s modulus. In this context, the subject of the current paper addresses longitudinal elastic modulus measurement for a small size sample made from Al_5_Cu_0.5_Si_0.2_Zn_1.5_Mg_0.2_, an experimental concentrated alloy complex (CCA). The complex concentrated alloy, derived from high entropy alloy concept (HEA) [24], is a metallurgical concept based on the assumption that the existence of a large number of main elements allows for easier control of the solid solution structures [25]. Even if the interest for this new class of alloys is high [26,27,28,29,30], given their potential in terms of mechanical and thermal properties, in the development phase, the small size ingots do not allow classical static tests to determine the longitudinal modulus of elasticity

Therefore, in this work, we intend to measure Young’s modulus of metallic alloys by using long and thin samples and a classic SHPB. The proposed algorithm exclusively uses the signal recorded in the transmitted bar and its multi-step specific shape. In the mathematical processing of the acquired data, the existence of finite jump times, from one step to another, is admitted and used in calculations.

## 2. Theory

### 2.1. Elastic Longitudinal Wave Propagation in Bars

When an external force is applied to a body, internal deformations and stresses occur, both in the vicinity and remotely from the force application area. However, internal stresses that occur remotely cannot be transmitted instantly. Thus, with finite speed, state and motion disturbances propagate through the body in the form of waves.

The study of the elastic wave propagation in long bars, in terms of elastic strains, leads to relationship [31]:

(1) $$c_{0}={\left(\frac{d\sigma /d\varepsilon }{\rho }\right)}^{1/2}=\sqrt{\frac{E_{a}}{\rho }}$$

where $c_{0}$ is the speed of sound, σ stands for bar axial stress, ε indicates bar axial strain, ρ denotes bar density, and *E_a_* represents the bar elastic modulus.

The thermodynamic transformation triggered in the environment by a longitudinal elastic wave is adiabatic and isentropic. For this reason, the modulus of elasticity expressed by (1) differs in value from the isothermal modulus of elasticity, *E_i_*, which is considered to be specific to static and quasi-static test methods [1]. The mismatch is due to the fact that the adiabatic transformation implies a change in the environment temperature, respectively, of the elastic strain as a result of the dilatation of the environment [32]. For most metals this difference is about 0.5% [1].

### 2.2. Influence of Impedance Mismatch on the Longitudinal Wave Propagation in Bars

The product $\rho c_{0}$ has a constant value for a given material and is called acoustic impedance. In some technical applications, such as the split Hopkinson pressure bar, where the tested sample is positioned between two identical bars, longitudinal elastic waves are generated by impact and pass bodies with different cross sections and acoustic impedances.

The product between the acoustic impedance and the cross-section area is called the mechanical impedance, *Z*, and is defined as the ratio between the driving force, *F*, and the material velocity, *u_m_* [33]:

(2) $$Z=\frac{F}{u_{m}}=A\rho c_{0},$$

where *A* is the cross-section area.

Due to the specific SHPB mechanical impedance discontinuity, a sudden shift from the bar mechanical impedance $A\rho c_{0}$ to the sample mechanical impedance $A^{\prime }\rho^{\prime }c_{0}^{\prime }$, a process of reflection and partial transmission of the wave on the incident bar/sample interface, is exhibited. The process is repeated on the sample/transmitter bar interface, the waves traversing the sample back and forth until the elastic pulse is ended.

Thus, at the first back and forth pass of the elastic wave through the sample, only a part of the incident stress, $\sigma_{i}$, fraction $\sigma_{t}$, is passed to the transmitter bar. The rest of the stress, $\sigma_{r}$, is reflected in the incident bar as a rarefaction wave that overlaps the incident wave.

To quantify stresses that are reflected and transmitted at the interfaces, it is necessary to understand the dynamics of each interface. At each sample/bar contact interface, the velocities of the two bodies are equal, as long as they remain in contact during the test. Moreover, the forces on the left and those on the right of the interface must be equal to satisfy the equilibrium condition. By imposing these two conditions, we can write the equations that describe the effects of interfaces on wave propagation. The systems of equations for these two interfaces, for the first back and forth pass of the elastic wave into the sample, are the following:

- For the incident bar/sample interface

(3) $$\left\{ \begin{array}{l} \frac{\sigma_{i}-\sigma_{r}}{\rho c_{0}}=\frac{\sigma_{t}^{\prime }}{\rho^{\prime }c_{0}^{\prime }}\\ A(\sigma_{i}+\sigma_{r})=A^{\prime }\sigma_{t}^{\prime } \end{array}\right.$$

- For the sample/transmitter bar interface

(4) $$\left\{ \begin{array}{l} \frac{\sigma_{t}^{\prime }-\sigma_{r}^{\prime }}{\rho^{\prime }c_{0}^{\prime }}=\frac{\sigma_{t}^{}}{\rho c_{0}}\\ A^{\prime }(\sigma_{t}^{\prime }+\sigma_{r}^{\prime })=A\;\sigma_{t}^{} \end{array}\right.$$

where $\sigma_{t}^{\prime }$ is stress transmitted in the sample at the incident bar/sample interface and the $\sigma_{r}^{\prime }$ stands for stress reflected in the sample at the sample/transmitter bar interface.

By defining the transmission coefficient α, as a ratio between the incident waves and the transmitted wave,

(5) $$\alpha =\frac{\sigma_{t}}{\sigma_{i}}$$

the following values are obtained for these two interfaces:

(6) $$\alpha_{1}=\frac{2A\rho^{\prime }c_{0}^{\prime }}{A\rho c_{0}^{}+A^{\prime }\rho^{\prime }c_{0}^{\prime }}$$

(7) $$\alpha_{2}=\frac{2A^{\prime }\rho^{}c_{0}^{}}{A\rho c_{0}^{}+A^{\prime }\rho^{\prime }c_{0}^{\prime }}$$

where index 1 identifies the incident bar/sample interface and index 2 identifies the sample/transmitter bar interface.

Thus, at the first pass of the elastic wave through the sample/transmitter bar interface in the transmitter bar a stress occurs, $\sigma_{t1}$, given by the formula:

(8) $$\sigma_{t}{}_{1}=\alpha_{1}\alpha_{2}\sigma_{i}$$

If the duration of the elastic pulse is longer than the time required for the elastic wave to travel the sample back and forth, the reflection and transmission phenomena are repeated at these two interfaces. Based on the same conditions used to write (3) and (4), the stress reached in the second bar can be calculated after *n* passes of the wave through the sample/transmitter bar interface with the formula:

(9) $$\sigma_{t}{}_{n}=\alpha_{1}\alpha_{2}\sigma_{i}\left(1+h+\cdots +{\left(h\right)}^{n-1}\right)$$

where

(10) $$h={\left(\frac{A\rho c_{0}^{}-A^{\prime }\rho^{\prime }c_{0}^{\prime }}{A\rho c_{0}^{}+A^{\prime }\rho^{\prime }c_{0}^{\prime }}\right)}^{2}$$

represents the square of the ratio between the difference and the sum of the mechanical impedances of the bars and the tested sample.

**Figure 1:**
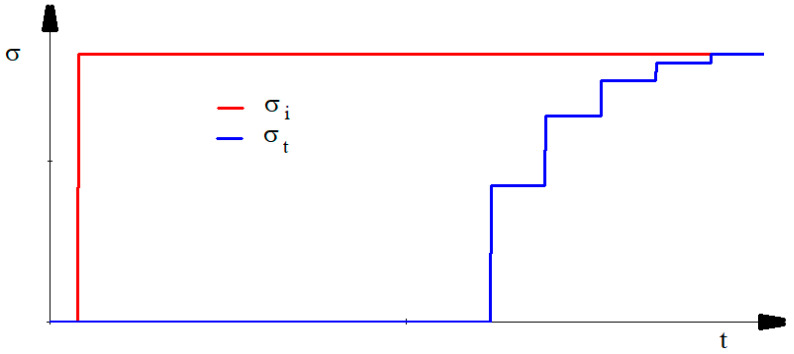
Transformation of a single-step elastic pulse into a multi-step elastic pulse when passing through a sample with a different mechanical impedance than the bars.

Consequently, the evolution of the stress in the transmitter bar, under the conditions of a step-like shape of the initial elastic pulse, in which the value of the incident stress $\sigma_{i}$ remains constant throughout the pulse, is presented as a multi-step signal, Figure 1. The ratio between two successive steps is equal to the subunit value h as it results from the relation:

(11) $$\frac{\sigma_{t}{}_{n}-\sigma_{t}{}_{n-1}}{\sigma_{t}{}_{n-1}-\sigma_{t}{}_{n-2}}-=h$$

Processing the recorded data from such a test is limited to the determination of the dimensionless value *h* which faithfully reproduces the ratio between the heights of two successive steps from the signal recorded by a strain gauge placed on the transmission bar. For this reason, the classic data processing procedures used in SHPB tests are not necessary. Using (10) and the value of the experimentally determined constant *h*, the modulus of elasticity of the sample is calculated as:

(12) $$E^{\prime }={\left(\frac{1-\sqrt{h}}{1+\sqrt{h}}\right)}^{2}\frac{A^{2}\rho^{2}c_{0}^{2}}{A^{\prime }{}^{2}\rho^{\prime }}$$

**Figure 2:**
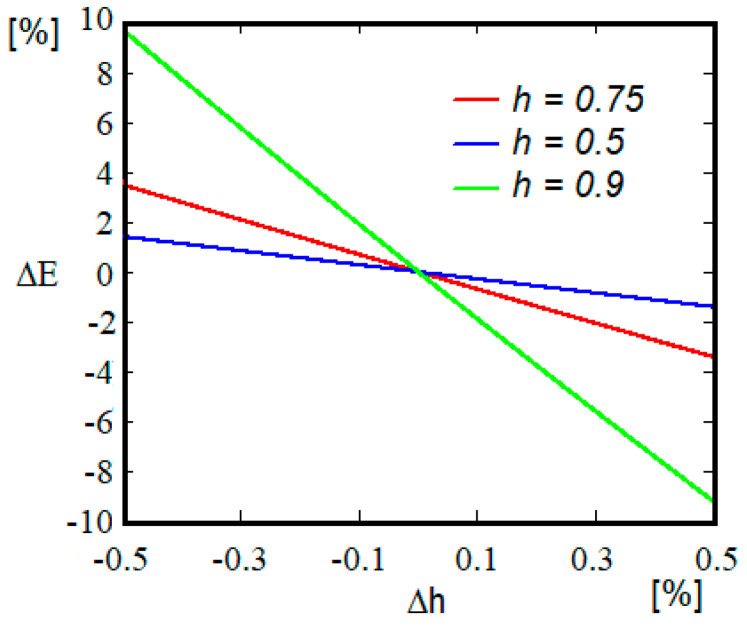
Influence of the measurement error Δh on the value longitudinal modulus.

An analysis of the sensitivity of the Young’s modulus calculated value to the measurement errors of this parameter is required. Figure 2 shows the variations of the Young’s modulus according to the variations of the parameter *h* for three distinct values. It is observed that as the value of *h* increases, the measurement errors have a greater impact on the calculated value of the Young’s modulus.

Regarding the characteristic time necessary for the elastic wave to travel back and forth on the sample, this is calculated with the following formula:

(13) $$\tau^{\prime }=\frac{2l^{\prime }}{c_{0}^{\prime }}$$

where $l^{\prime }$ is the sample length.

The rewriting of the relation (13) allows expressing the sound velocity in the sample according to the characteristic time and the sample length:

(14) $$c_{0}^{\prime }=\frac{2l^{\prime }}{\tau^{\prime }}$$

which finally leads to express the elasticity of the sample as

(15) $$E^{\prime }=\rho^{\prime }{\left(\frac{2l^{\prime }}{\tau^{\prime }}\right)}^{2}$$

## 3. Experimental Setup and Numerical Model

### 3.1. Tested Materials

The tests were performed on an Al_5_Cu_0.5_Si_0.2_Zn_1.5_Mg_0.2_ alloy specimen with a diameter of 8.20 mm and a length of 68.7 mm, manually placed between the bars. The 3800 kg/m^3^ density complex concentrated alloy was obtained by melting higher purity elements (purity > 99.5%) in a laboratory induction furnace type Linn MFG-30 (Linn High Therm GmbH, Eschenfelden, Germany), with a controlled atmosphere. The alloying process was performed at 700–800 °C and 0.6 ÷ 0.8 bar in argon atmosphere, and the melting crucible used was made of alumina. The prepared alloy was then cast in a copper mold under protective atmosphere. The chemical composition of the resulted specimens was determined by the ICP-OES method. The results obtained are presented in Table 1. The chemical analysis shows a resulted composition close to the nominal composition of the alloy without significant differences.

Al_5_Cu_0.5_Si_0.2_Zn_1.5_Mg_0.2_ is a complex concentrated alloy that contains elements in higher proportion than the conventional aluminum alloys. Al_5_Cu_0.5_Si_0.2_Zn_1.5_Mg_0.2_ was selected based on the inexpensive combining elements, ease of formation of solid solutions with light elements, high corrosion resistance of the elements, and potential for increased mechanical resistance. Optical analyses of the Al_5_Cu_0.5_Si_0.2_Zn_1.5_Mg_0.2_ alloy shows a fine dendrite structure with a number of 3 major phases and 2 eutectic structures. The percentage of solid solutions is higher than the inter-metallic compounds.

### 3.2. SHPB Equipment

The SHPB system used for tests has 20 mm diameter steel bars of 7800 kg/m^3^ density and 210 GPa elasticity modulus. The projectile has a length of 385 mm and the bars a length of 2000 mm. The strain gauge bridges were mounted at 800 mm on the bars ends. The signals generated at the Wheatstone bridge strain gauge were conditioned with Ectron 778 signal conditioners (Ectron Corporation, San Diego, CA, USA) and recorded with a 6000 series Picoscope (Pico Technology, St Neots, UK). In order to attenuate the high frequencies that occurred at the impact, the end surface of the projectile was covered with a paper adhesive band.

### 3.3. Numerical Model

**Figure 3:**
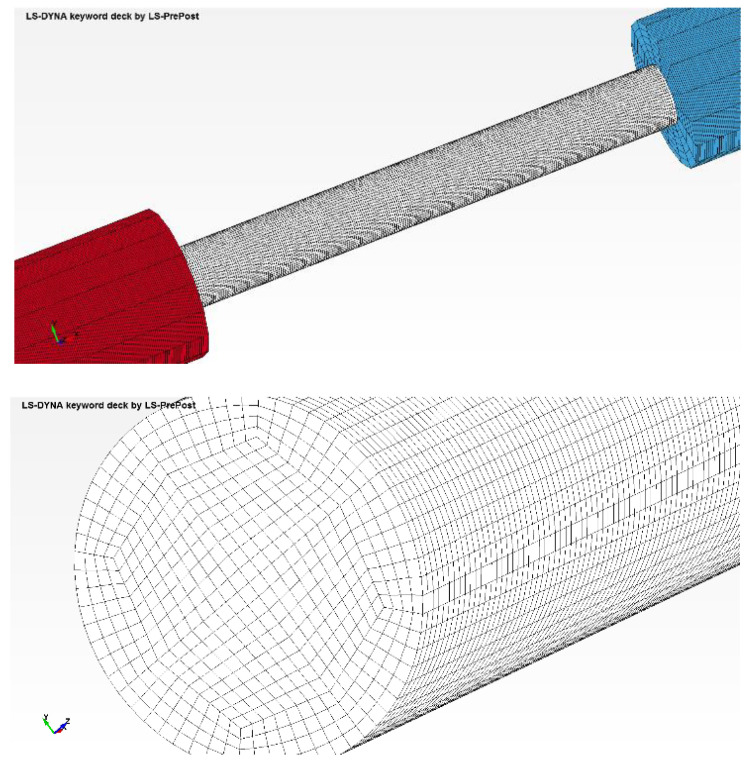
Detail view of the virtual model on the region of the sample (**up**) and nodes arrangements in the sample cross section (**down**).

In order to verify the validity of previously-stated hypotheses for the elastic modulus evaluation, a Ls-Dyna numerical simulation was performed. The numerical model geometry and mesh are detailed in Figure 3.

For the mesh as depicted in Figure 3, a hex shape solid element was used (type SOLID 1—constant stress solid element).

For higher accuracy, the numerical model was set-up considering the following aspects:

➢ A 3D modeling approach based on the real dimensions of the bars and samples, and also the real initial and boundaries conditions, is preferable. Both incident and transmitted bars are 20 mm in diameter and 2000 mm in length. The striker length is 385 mm. The specimen has 67.8 mm length and 8.2 mm diameter.
➢ A fine mesh must be imposed for the longitudinal direction in order to capture the real phenomena. Thereby, a 0.25 mm length mesh was considered, a value smaller than the one usually used in mesh sensitivity analyses for similar SHPB tests [21,34,35]. The number of elements and nodes of every component of the SHPB are presented in Table 2.
➢ The contact between parts must be as real as can be simulated which leads to the use of the *CONTACT_AUTOMATIC_SURFACE_TO_SURFACE_ID card. For the initial striker velocity, the *INITIAL_VELOCITY_GENERATION card was used.

Linear elastic constitutive behaviors were assigned to the bars and specimens. The Hopkinson bars are made of steel with the following material and geometric characteristics: elastic modulus—210 GPa, Poisson’s ratio—0.3, density—7800 kg/m^3^, diameter—20 mm, and length—2000 mm for both incident and transmitted bars. The striker length is 385 mm and the specimen has 67.8 mm length and 8.2 mm diameter.

## 4. Results

### 4.1. Measured Data

**Figure 4:**
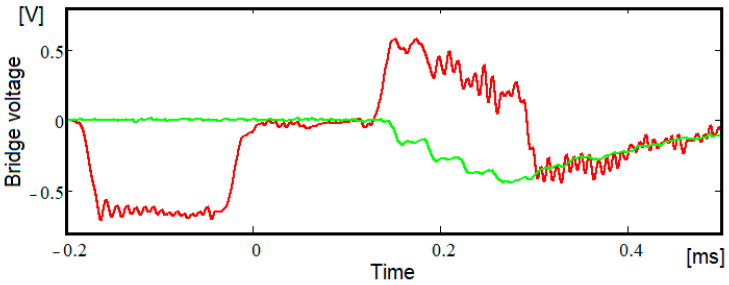
Typical signal recorded during the tests with Picoscope software.

The typical signals recorded during the tests, for both bars, are given in Figure 4. The red curve represents the signal acquired by the Wheatstone bridge from the incident bar and the green one represents the signal acquired by the Wheatstone bridge from the transmitter bar. The multi-stepped feature of the transmitter bar predicted by Equation (9) is present. Still, the real signal is not completely similar to the ideal signal from Figure 1. Firstly, the time necessary to rise from a level to the next one is finite, a characteristic observed in tests of a similar setup [23]. Secondly, the signal is oscillating on the segments where it should be flat, which is proof of the existence of high-frequency harmonics.

A better highlight of the existence of the threshold is obtained when several tests performed on the same sample are overlapped repeatedly.

**Figure 5:**
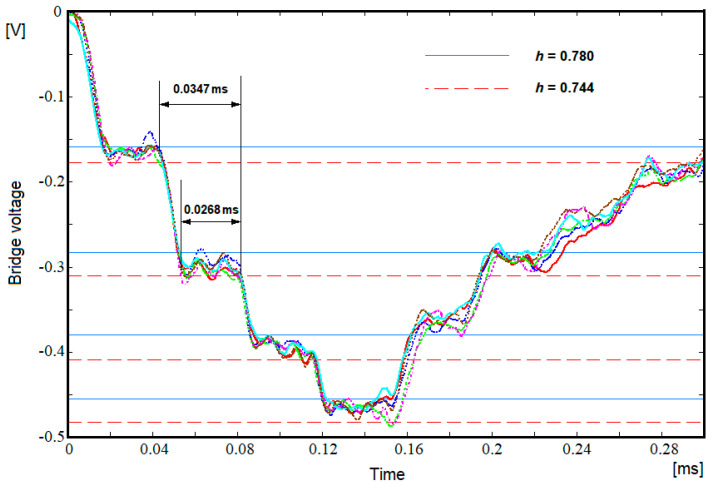
Signals recorded with the Wheatstone bridge from the transmitter bar for the tests on the long sample.

The signals recorded during the tests in the transmission bar, after scaling, are shown in Figure 5.

The scaling was necessary because the impact velocities were similar, but not identical, and allowed the deformations in the sample to be kept below the threshold occurrence of plastic deformations. The occurrence of elastic deformation was checked by measuring the sample length after each test. Simultaneous plotting of the signals clearly shows the existence of thresholds in the signals. At the same time, it is observed that due to the oscillations presence we can actually see an interval in which *h* can vary and for which the thresholds drawn on the basis of *h* overlap with the horizontal signal segments. The same figure shows the thresholds for two values of *h*, 0.78 and 0.744, which roughly represent the limits of this interval. Based on these two values, using the Formula (12), the elastic modulus can be calculated being obtained as59 GPa, respectively, 83 GPa, with the difference between the two being unacceptable.

### 4.2. Methodology for Refining the Value of the Modulus of Elasticity

However, in order to refine the solution, we also have the characteristic time necessary for the elastic wave to traverse the sample back and forth given by Formula (13). Thus, an analysis of the real signals superimposed in Figure 5 indicated that the time difference between the starting moment of one level and the starting moment of the next level is approximately constant for all three jumps. An approximate average value of 0.0347 ms is obtained. If we admit that the previously indicated time intervals could represent the characteristic time of the wave passing the sample back and forth and using Formula (13), in which the value of 67.8 mm is introduced as the length of the sample, we obtain for the sound velocity the value of 3910 m/s, which corresponds to a modulus of elasticity of 58.1 GPa and a dimensionless characteristic value *h* of 0.781, a value that is found outside the range 0.744 to 0.78. This situation indicates that the characteristic time to be used in Formula (13) is shorter.

An additional analysis of Figure 5 shows that the jumps from one level to another are made in time intervals of about 0.0079 ms and can be approximated with straight lines with different inclinations, which is why the quasi-horizontal signal segments have approximately the same length in time, of about 0.0268 ms, as seen in Figure 5. If one admits that the previously established time intervals could represent the time needed by the elastic wave to move back and forth through the sample and considering Formula (13) as custom for a 67.8 mm sample length, a result of 5060 m/s value for the sound velocity corresponding to a modulus of elasticity of 97.3 GPa and a dimensionless characteristic value *h* of 0.726 (a value that is found outside the range 0.744–0.78) is achieved. This result indicates that the characteristic time that must be used in Formula (13) is longer.

**Figure 6:**
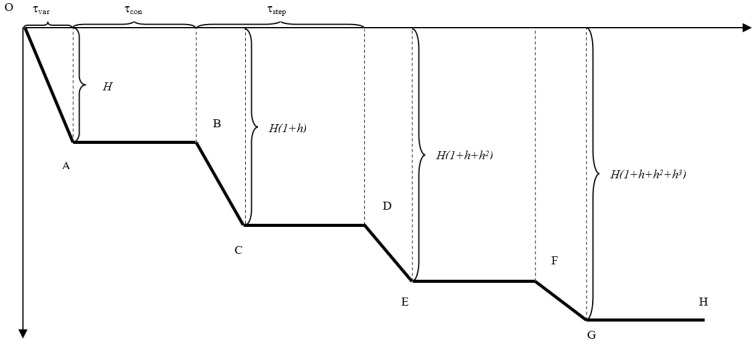
Schematic signal given by the branches function *f*(*x*).

However, the admission of the existence of a fixed value for the rise time from one level to another leads to a constant ratio of the slopes of the lines that schematize two successive rises, with the value being also given by the Formula (10). In other words, the schematization of the signals by succession of horizontal lines and inclined lines is controlled by two dimensionless parameters, *h* and *H*, and by two temporal parameters, the rise time *τ_var_* and the step time *τ_step_*, which are found depending on the branches (16) which describe the graph in Figure 6. The admission of Formula (16) also assumes the existence of a characteristic time *τ_const_ =* *τ_step_ −* *τ_var_*

(16) $$f\left(x\right)=\{\begin{array}{c} x\frac{-H}{\tau_{var}},0\leq 0\leq \tau_{var}\\ -H,\tau_{var}<x<\tau_{step}\\ x\frac{-Hh}{\tau_{var}}+\frac{\left(\tau_{step}-\tau_{var}\right)\left(H\left(1+h\right)\right)+\tau_{step}H}{\tau_{var}},\tau_{step}\leq x\leq \tau_{step}+\tau_{var}\\ -H\left(1+h\right),\tau_{var}+\tau_{step}<x<2\tau_{step}\\ x\frac{-Hh^{2}}{\tau_{var}}+\frac{\left(2\tau_{step}-\tau_{var}\right)\left(H\left(1+h+h^{2}\right)\right)+2\tau_{step}H\left(1+h\right)}{\tau_{var}},2\tau_{step}\leq x\leq 2\tau_{step}+\tau_{var}\\ -H\left(1+h+h^{2}\right),\tau_{var}+2\tau_{step}<x<3\tau_{step}\\ x\frac{-Hh^{3}}{\tau_{var}}+\frac{\left(3\tau_{step}-\tau_{var}\right)\left(H\left(1+h+h^{2}+h^{3}\right)\right)+2\tau_{step}H\left(1+h+h^{2}\right)}{\tau_{var}},3\tau_{step}\leq x\leq 3\tau_{step}+\tau_{var}\\ -H\left(1+h+h^{2}+h^{3}\right),\tau_{var}+3\tau_{step}<x<4\tau_{step} \end{array}$$

It is obvious that the function admits a generalization for the case of the existence of a certain number of *n* jumps.

To determine these four parameters based on an existing signal, the following methodology was defined: for the second and third jumps, the inclined lines that best approximate them are calculated using the least square method. Dividing the slope of the line corresponding to the third jump to the slope of the line corresponding to the second jump, the value of *h* is obtained. This information is sufficient to calculate the elastic modulus in Formula (12) and the sound velocity by which the characteristic time is calculated.

The signal part corresponding to the last level is determined with the help of the least squares method and the horizontal line, GH, which best approximates it. It is situated at a distance $H\left(1+h+h^{2}+h^{3}\right)$ from the horizontal. Thus, parameter *H* can be calculated.

Drawing a horizontal line at a distance *H* from the horizontal (the first level) allows the determination of point B: the intersection point between the horizontal and the line that approximates the second jump. Drawing the horizontal line at the distance H(1+h) allows to determine point C (the intersection point of the second level with the line approximating the second jump) but also to determine point D (the intersection point of the second level with the line approximating the third jump). The time distance between points B and C represents the characteristic rise time and the distance between points B and D represents the characteristic time of a cycle. The determination of these characteristic times allows a subsequent determination of points O, A, E, F, and G, and the plotting of the schematic signal for all four jumps. If the schematic signal shows significant differences from the real signals, an adjustment for the *h* value is required for a better match.

**Figure 7:**
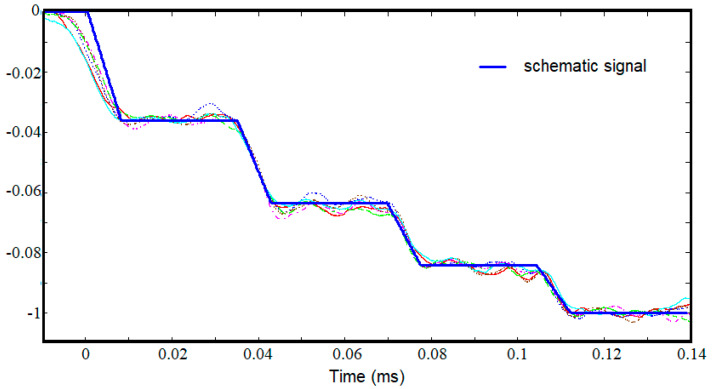
Schematic signal superimposed on experimental signals.

For the experimental data available, the following values were determined, *h* = 0.758, *τ_var_* = 0.0085 ms, and *τ_step_* = 0.0346 ms. For these values, the schematic signal was built up and superimposed over the experimental signals, as presented in Figure 7. The representation is normalized so that the last jump of the schematic signal is at a distance of one unit from the axis 0. As can be seen, the signal schematic accurately reproduces the experimental signals except for the first jump, which corresponds to the first passage of the elastic wave through the sample.

The longitudinal elastic modulus calculated with Formula (12) has the value 73 GPa and the characteristic time *τ* given by Formula (13) is 0.0309 ms.

### 4.3. Numerical Results

For the tested material, only the density (3800 kg/m^3^) and the longitudinal elastic modulus (73 GPa) are known as they had been presented or determined in the previous sections. In order to fully state an elastic material model, it is also necessary to define the Poisson’s ratio. Since the exact value of this parameter is unknown, several simulations were performed in which for the Poisson’s ratio the values 0.26, 0.28, 0.3, 0.32, and 0.34 were assigned.

**Figure 8:**
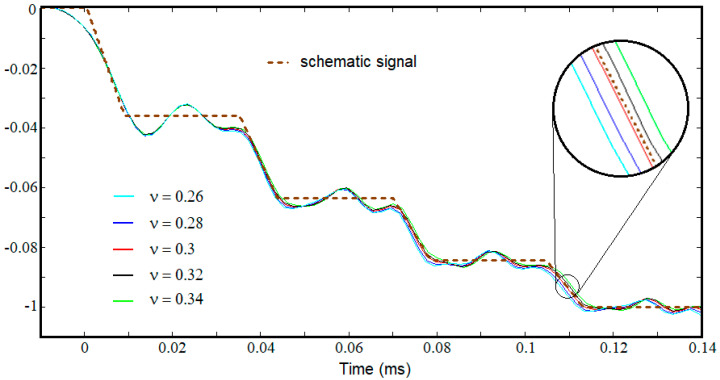
Comparison of schematic signal with the simulations results.

The signals recorded by the virtual transducer placed on the same coordinates as of the Wheatstone bridge used in the experiment on the transmission bar are shown in Figure 8 along with the schematic signal obtained based on the real tests. It is clear that the differences between the overlapping signals are imperceptible in terms of threshold values. Instead, there are differences regarding the time values when the jumps occur, differences that increase from one jump to another. This result highlights the influence that the Poisson’s ratio value has on the characteristic times *τ_var_* and *τ_step_*. The two characteristic times vary inversely with the Poisson’s ratio for a constant value of the longitudinal elastic modulus.

The simulation that succeeds to reproduce, with the highest fidelity, both the ratios between the successive thresholds and the time durations of the signal segments corresponding to the jumps and levels is the one in which the Poisson’s ratio was considered 0.3.

The numerical results are similar to the experimental ones which confirm the validity of the used hypothesis considered for the construction of the schematic signal, namely, constant jump times *τ*_var_.

**Figure 9:**
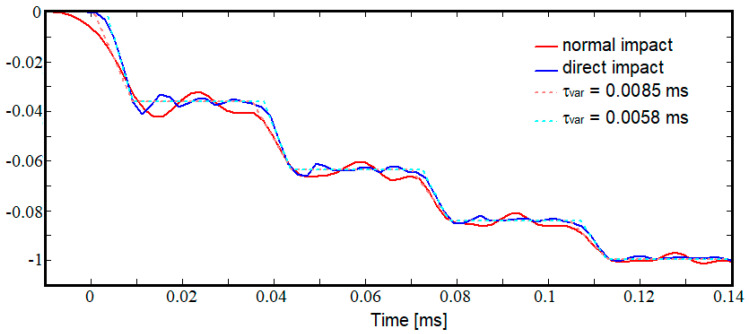
Comparison between the simulations results for the standard impact and direct impact.

As Figure 9 highlights, the overlap of the real and numerical signals for the first jump of the signal is poor. For a further investigation of this discrepancy, a simulation in which the incident bar impacts the sample directly was performed. The signal from the transmission bar was collected from an element displaced at 5 cm from the sample–transmission bar interface. For the recorded signal, the methodology for determining the schematic signal was applied and ended in the following results: *h* = 0.758, *τ_var_* = 0.0058 ms, and *τ_step_* = 0.0346 ms. In Figure 9, the signals recorded for the standard and the modified configuration along with the two schematic signals are plotted. As can be seen, the signal fit for the modified configuration case is also achieved for the first jump. This highlights the fact that the matching problem for the first jump, observed both in the experimental and standard configuration cases, is no longer an issue when the elastic pulse is generated directly by the striker on the tested sample.

The direct impact also eliminates the dispersion of the train of elastic waves during wave propagation through the bars before acting on the sample and reaching the section where the gauge was mounted.

Moreover, it is observed that even in the case of direct impact, the characteristic time *τ_step_* 0.0288 ms obtained for the schematic signal does not correspond to the characteristic time $\tau^{\prime }$ given by Formula (13) for a modulus of elasticity of 73 GPa.

## 5. Discussions

The work of other researchers on this topic has focused mainly on assessing deviations from the ideal assumptions generally used by the mathematical apparatus developed for SHPB. To overcome these shortcomings, the FEM analysis and samples with complicated shapes and accessorized with strain gauges were used [21,22].

Compared with the above, our approach is based on theoretical relations related to the propagation of elastic waves in bars in the conditions of a sudden change of mechanical impedance. Those relations lead to a mathematical equation, Formula (12), that shows the connection between the adiabatic elastic modulus of the sample and *h*, the ratio between the heights of two successive steps which occur when long and thin samples are used. However, the pattern of real signals differs from the ideal one, with the presence of oscillations making it difficult to determine exactly the value of *h* by using only Formula (12). To overcome the existence of oscillations on the signals for the finite rise time of each step, another inherent feature of real signals [23] can be used. By assuming that there is a signal pattern in which not only the constant signal segments but also the jump segments have the same durations at each step, *τ_const_* and *τ_var_*, a refined value of *h* and, consequently, a refined value of Young’s module can be calculated.

A really interesting finding is the one related to the calculated value of characteristic time $\tau^{\prime }$ necessary for the elastic wave to travel back and forth on the sample, given by Formula (13) and mentioned in [23]. It has been shown that neither of the two characteristic times, *τ_step_* and *τ_const_*, represent this characteristic time. This result shows that it is not possible to clearly delimit on the acquired signal which is the portion of time that corresponds to the wave passing back and forth through the sample.

On the other hand, the fact that the sample is not in tight contact with the bars at the beginning is a possible explanation for the difference between the schematic signal and the real one observed at the first jump, Figure 7.

The results of the tests and the FEM simulations performed so far prove the validity of the stated hypotheses on which this methodology was built. Moreover, as long as the complex concentrated alloy Poisson coefficient was not determined experimentally, the simulations were used for a supplementary investigation on the influence of the Poisson coefficient on the characteristic times. It was shown that the *τ_step_* and *τ_var_* times increase when the coefficient value decreases; however, the influence is small and cannot be expressed by analytical relations usable in the calculation of this coefficient.

It is necessary to observe that the possible errors in the measurement of the ratio *h* are reflected in the final value of the modulus of elasticity, with the sensitivity increasing with the value of *h*, Figure 2. Still, it is not recommended to use samples that lead to low values of *h* because small steps can no longer be detected due to the existence of oscillations in the signal.

Given the above, the main limitation of the method is the natural occurrence of the wave scattering phenomenon during the propagation of the elastic pulse along the bars which may affect the accuracy of the calculated value. Based on the direct impact simulation results, there are reasons to consider the testing method suitable for accuracy improvements. Moreover, the theoretical relationships for SHPB torsion tests indicate that the transverse modulus of elasticity *G* can be determined in a similar way to Young’s modulus. Last but not least, the possibility of applying the presented method in the case of bar-type samples with a square or hexagonal cross section or made from orthotropic materials, such as composites, is of interest.

It can be concluded from the above discussions that our proposed method has the advantage of using existing laboratory equipment in a simple way, being an affordable alternative to existing test methods.

## 6. Conclusions

After conducting the SHPB compression tests for a long and thin metallic sample it was confirmed that this type of test may provide experimental data that can be exploited to accurately calculate the Young’s modulus of the tested material. The main findings of the results analysis are summarized below:

(a) Despite the deduction of an analytical calculation in Formula (12), the real aspect of the acquired data prevents its reliable use;
(b) The accurate calculation algorithm developed on the basis of a schematic signal, which is much closer to the real shape of the acquired signal, admits the existence of a finite jump time from one level to another, *τ_var_*;
(c) The results of the tests and the FEM simulations performed so far prove the validity of the stated hypotheses on which this methodology is built;
(d) There is a certain potential for improving the method as well as the possibility to be used in determining the transverse modulus of elasticity.

## Figures and Tables

**Table 1:** Complex concentrated alloy composition.

Alloy Al_5_Cu_0.5_Si_0.2_Zn_1.5_Mg_0.2_	Composition, wt. %				
	Al	Cu	Si	Zn	Mg
Nominal	49	11.54	2.05	35.64	1.77
Experimental	43.86	11.39	2.02	38.66	2.02

**Table 2:** Mesh characteristics.

SHPB Component	Elements	Nodes
Striker	80,192	94,640
Incident bar	391,040	463,097
Sample	138,752	148,240
Transmitted bar	391,040	463,097

## Data Availability

The raw/processed data required to reproduce these findings cannot be shared at this time as the data also forms part of an ongoing study.
